# Environmental Tax Reform and the “Double Dividend” Hypothesis in a Small Open Economy

**DOI:** 10.3390/ijerph17010217

**Published:** 2019-12-27

**Authors:** Zhibo Zhou, Weiguo Zhang, Xinxin Pan, Jiangfeng Hu, Ganlin Pu

**Affiliations:** 1 College of Economics and Management, Southwest University, Chongqing 400715, China; xmuzzb@163.com (Z.Z.); zwg@swu.edu.cn (W.Z.); pxxbook1@email.swu.edu.cn (X.P.); leisure@email.swu.edu.cn (J.H.); 2 School of Political Science and Public Management, Southwest University, Chongqing 400715, China; 3 School of Finance, Zhongnan University of Economics and Law, Wuhan 430073, China

**Keywords:** environmental taxes, double dividend hypothesis, small open economy, employment, economic growth

## Abstract

In this paper, we build and analyze a general equilibrium model to evaluate the effects of environment tax reform on a small open economy in a “suboptimal environment” with existing tax distortions. We then use the macroeconomic data from the Chongqing Municipality in China to conduct simulations to empirically test our analytic results. Our main findings include the followings. First, an increase in environmental tax rate can effectively reduce the use of polluting consumer goods by households as well as investment in polluting factors by enterprises. Hence, an increase in environmental tax rate can improve environmental quality and obtain “environmental dividend”. Second, an increase in environmental tax rate can negatively impact employment, family income and economic growth. Hence, there is no “non-environmental dividend” effect. Third, an increased environmental tax rate has both substitution effect and income effect on household consumption. On the one hand, it motivates households to substitute polluting consumer goods with clean consumer goods. On the other hand, it lowers the total consumption level of households. Fourth, we show that the “double dividend” hypothesis on environmental tax is invalid. And the optimal environmental tax under the suboptimal environment is lower than the Pigouvian tax rate. Finally, we discuss the policy implications of our results.

## 1. Introduction

Achieving sustainable growth and improving people’s health and standard of living have always been the two major tasks for any government [1]. To this end, more considerations have been given to the use of environmental regulations, including environmental taxes. Earlier studies on the impacts of environmental tax and its reform are mostly based on the assumption of perfectly competitive market [2,3]. These studies tend to support the “double dividend” hypothesis [4,5]. That is, environmental tax reforms will improve economic efficiency and environmental quality simultaneously. The improvement of environmental quality is often called “the first dividend” or “the environmental dividend”, while the improvement of economic efficiency in terms of employment, growth, and income is often called “the second dividend” or the “non-environmental dividend”. According to the nature of the non-environmental dividend, “double dividend” can categorized further. Goulder [5] proposed to categorize “double dividend” into weak form and strong form. In the literature, most early empirical studies supported the weak double dividend hypothesis [5,6]. The strong form of double dividend is mostly found in the Nordic countries because of their labor market distortion and greater benefit from the reform.

However, after the introduction of computational general equilibrium (CGE) models in the literature, more normative research tends to confirm the weak double dividend hypothesis [6]. The query mainly comes from the following aspects. First, the environmental tax does not necessarily improve the environmental quality. Second, it is difficult for the government to set the environmental tax rate optimally. Third, the use of environmental tax revenues for structural tax reduction is not necessarily more efficient than the use of a total tax return (lump-sum transfer). Since then, the support of the strong double dividend hypothesis has been gradually weakened [7]. Many studies show that only under specific conditions, the strong double dividend hypothesis can be valid [8]. If more attention is paid to the impact of environmental tax on labor demand, the conclusions tend to support the double dividend hypothesis of employment [9,10]. However, if more attention is paid to the impact of environmental tax on the supply of labor, the conclusions usually negate the double dividend of employment. Zhou and Zhang [11] pointed out that the existing research on the double dividend which concludes that environmental tax is more likely to promote economic growth is usually done in mature market economies.

For the study of double dividend effect of tax burden, Poterba and Rotemberg [12] proposed to measure the regressiveness (or progressiveness) of environmental taxes by calculating the proportion of household energy (fuel) expenditure in family income. If the proportion in low-income families exceeds that of the high-income families, the environmental tax can be considered regressive, and there is no double dividend effect of tax burden. Since then, this method has been widely adopted, and most studies support that the environmental tax usually benefits wealthy families and negates the double dividend of tax burden. Moreover, research on the double dividend of allocation usually shows that, whether in terms of horizontal income distribution or vertical welfare distribution, environmental taxes tend to be advantageous to the wealthier and aggravate income inequality [13]. In other words, there is no double dividend effect of distribution [14]. Furthermore, several studies point out that the environmental tax will inevitably cause unfair distribution among different generations [15,16,17].

In this paper, we examine the economic effects of environmental taxes in a small open economy. We first build and analyze a general equilibrium model and obtain analytic results. To further verify these results, we use the macroeconomic data of the Chongqing Municipality in China to conduct simulations on environmental tax reform. A small open economy is a suboptimal environment where environmental distortions and tax distortions may exist. Environmental distortions mean that firms and individuals ignore the negative externalities of environmental pollution when making production and consumption decisions although environmental pollution in turn affects firm profits and utilities. Tax distortions mean that lump-sum tax is infeasible. The government must rely on the distortion of the tax revenue for the public expenditure [18]. In this article, the distortionary tax mainly refers to the existing taxes closely related to labor incomes in the economy.

Here, it is worthwhile to explain why we study the policy effect of environmental tax reform on a small open economy, and why we choose the Chongqing Municipality of China for empirical simulations. The explanations and reasons are as follow. On the one hand, the environmental tax reform was initiated by developed countries in Europe, such as the Nordic countries. Most of these countries can be regarded as small open economies. The research stream on environmental tax started mostly based on data from small open economies, and then gradually relaxed the relevant economic assumptions. Hence, this research follows this tradition. On the other hand, as one of the four municipalities directly under the central government in China, Chongqing’s economy is to a great extent in line with the characteristics of a small open economy. That is, it has an insignificant impact on the global economy, but it is sufficiently open so that various elements can flow across regions with few restrictions.

In our model, environmental tax revenue is assumed to be used for structural reduction of pre-existing distortionary taxes in the economy, so as to maintain the overall stability of fiscal revenue. This is the so-called “fiscally neutral” principle. Our results derived from model analysis show that fiscally-neutral environmental tax reform can reduce the environmental distortion and improve the environmental quality. On the other hand, environmental tax reform can aggravate the distortion of the whole tax system. Hence, there is a trade-off between the two conflicting goals of environmental protection and the efficiency of the tax system. Moreover, our results indicate that the optimal environmental tax level under a suboptimal environment is lower than the Pigouvian tax rate. Furthermore, our simulations show that compared with firm environmental tax (i.e., the tax imposed on firms), family (household) environmental tax (i.e., the tax imposed on families or households) can greatly improve environmental quality at a lower cost of employment and economic growth.

The rest of the paper is organized as follows: Section 2 presents the main model and derives analytic results. Section 3 analyzes the welfare effects of environmental tax reform and explains their economic implications. Section 4 presents the simulations based on Chongqing’s economic data so as to empirically examine the theoretical results derived earlier. Finally, Section 5 summarizes our research findings and makes policy suggestions.

## 2. Main Model

In the main model, we assume that there is only one representative family and one representative firm in the small open economy. The government only levies environmental tax on the intermediate inputs and consumer goods which pollute. Moreover, the price tax is levied on the wage income.

### 2.1. Model Assumptions

#### 2.1.1. The Behavior of the Representative Firm

The following four assumptions are made for the representative firm: (a) the firm only produces one product (Y); (b) the firm makes production decisions in accordance with the principle of “profit maximizing”; (c) the firm faces a perfectly competitive product market; (d) the firm uses two production factors--labor (L) and pollution factor (E), and its production function F(L,E) has the characteristics of constant returns to scale. The firm pays the market wage W to the worker, and the government levies an ad valorem tax $T_{L}$ on the wage (a value-added tax measured by the quantity of the product), and a specific duty $T_{E}$ on the polluting factor. The profit of the representative firm can be written as follows:

(1) $$\pi =P_{Y}Y-\left(1+T_{L}\right)WL-\left(P_{E}+T_{E}\right)E,$$

where $P_{Y}$ and $P_{E}$ are product price and pollution factor price, respectively. The first order conditions for profit maximization are:

(2) $$P_{Y}\frac{\partial Y}{\partial L}=\left(1+T_{L}\right)W,$$

(3) $$P_{Y}\frac{\partial Y}{\partial E}=P_{E}+T_{E},$$

We have verified that the second-order conditions are satisfied so that the first-order conditions are sufficient to obtain the optimal solutions. For the sake of length, throughout this paper we omit these second-order conditions in the main text.

The conditions of complete competition, constant returns to scale and maximization of profits determine that the economic profits of firms are zero. Hence, we have:

(4) $$P_{Y}Y=\left(1+T_{L}\right)WL+\left(P_{E}+T_{E}\right)E,$$

#### 2.1.2. The Behavior of the Representative Family

It is assumed that the utility of a representative family depends on five variables: clean consumption goods (C), contaminating or polluting consumption goods (D), public consumption goods (G), environmental quality (M) and leisure (V). We define clean goods (*C*) to be the goods causing little pollution during their production and/or use. On the other hand, we define polluting goods (D) as the goods causing pollution in their production and/or use. Suppose the family utility function U=u[g(V,q(C,D)),h(G,M)] satisfies the condition that leisure and environmental quality are weakly separable from private consumption products in the family utility function. The family only earn income through labor with budgetary constraint as:

(5) $$WL_{S}=P_{C}C+\left(P_{D}+T_{D}\right)D,$$

Among them, $P_{C}$ and $P_{D}$ denote the prices of C and D, respectively, $T_{D}$ is the specific duty from D, and $L_{S}$ is labor supply quantity, which can be regarded as negative leisure. The family’s time endowment is normalized to 1. By constructing a Lagrange function, we can obtain the following first-order conditions for family utility maximizing:

(6) $$u_{V}=\lambda W$$

(7) $$u_{C}=\lambda P_{C}$$

(8) $$u_{D}=\lambda \left(P_{D}+T_{D}\right)$$

#### 2.1.3. The Behavior of the Government

Assume that the government’s income comes from the value-added tax $T_{L}$ imposed on labor income, and the environmental taxes $T_{E}$ and $T_{D}$ imposed on the polluting input E and the contaminated consumption goods D, respectively. The government’s expenditure is fully used to provide G (with the price $P_{G}$) for public consumption goods, where G is an exogenous variable. The budget constraint of the government is as follows:

(9) $$P_{G}G=T_{L}WL+T_{E}E+T_{D}D$$

In this model, the effects of rising rate of the environmental tax imposed on the family and the firm are examined when the environmental tax revenue is returned through tax reduction measures on labor income. Therefore, $T_{L}$ is endogenous to ensure the post-balance of government budgets.

#### 2.1.4. Environmental Quality

In order to simplify the problem, it is assumed that the environmental quality M is related to the polluting input E and the polluting consumption goods D. That is, M=m(D,E). Moreover, M is negatively related to D and E. That is, $m_{D}<0,\;m_{E}<0$. If pollution can be exported through international trade, the link between domestic environmental quality and domestic production and consumption will be weakened.

#### 2.1.5. International Trade

It is assumed that all products and polluting inputs can be used for trade. Therefore, in a small open economy, the prices of products and inputs are determined by the exogenous market in the world market. In addition, it is assumed that the labor force is not free to flow internationally, and the wages are fully flexible. Therefore, the wage rate is determined by the domestic labor market and the labor market is clear, that is $L=L_{S}$. According to the Walras Equilibrium Rule, we combine the zero-profit conditions of the firm, the budget constraint of the family, and the budget constraint of the government. We can then derive the following condition of international balance of payments (the budget constraint of the whole economy):

(10) $$P_{Y}Y=P_{E}E+P_{D}D+P_{C}C+P_{G}G$$

### 2.2. Model Analysis: Logarithmic Linearization

Starting with an initial equilibrium, logarithmic linearization of the model is carried out to analyze the effects of an environmental tax on domestic environment and economy. We adopt two assumptions in our analysis. First, the prices of trade products and inputs are determined by the world market, and the domestic environmental tax reform will not affect these prices. Second, the supply of public consumption goods G is determined by the external fiscal policy. For clarity, in this paper, “~” is used to represent the relative change of the variable.

#### 2.2.1. Variables, Parameters, Proportions and Their Relations

Before conducting logarithmic linearization, it is necessary to define the variables, parameters as well as their relations. The variables and their meanings are listed in Table 1.

#### 2.2.2. Logarithmic Linearization of the Model

Processing the functions of production, household, firm and the government by logarithmic linearization, we can solve the model and obtain the results, which are summarized in Table 2. See Appendix A, Appendix B and Appendix C for the derivations.

## 3. Welfare Effects of the Environmental Taxes

### 3.1. The Impact of Environmental Taxes on Welfare

#### 3.1.1. The Marginal Excess Burden (MEB)

The marginal excess burden (MEB) is used to measure the welfare effects of environmental taxes. According to Keller [19], Mooij [20], Freire-González [21] and Freire-González and Ho [22], the excess burden of environmental taxes is calculated by compensating variation. Compensating variation (CV) is the transfer income that households must receive to maintain their initial utility when policy shocks occur. The excess burden hence measures the welfare loss that exceeds the government’s environmental tax revenue. A positive excess burden means that environmental tax leads to welfare loss, and the negative excess burden means that environmental tax brings welfare income. Since the excess burden is not reflected in the tax revenue, the additional cost it causes can be regarded as the hidden cost of public expenditure financing. The compensating variation (CV) makes the family’s utility unchanged after the impact of the policy. Therefore, we have:

(11) $$0=\mathrm{dU}=\frac{\partial u}{\partial V}dV+\frac{\partial u}{\partial C}dC+\frac{\partial u}{\partial D}dD+\frac{\partial u}{\partial M}dM.$$

where we utilize the implied condition dG=0. At the same time, utility maximization means that the partial differential on the right-hand side of the equation is equal to the product of the marginal utility of the income and the corresponding price. That is, $-\lambda WdL+\lambda P_{C}dC+\lambda \left(P_{D}+T_{D}\right)dD+\frac{\partial u}{\partial M}dM=0$, which uses the equation dL=−dV. Differentiating the family budget constraint represented by Equation (5), we can obtain:

(12) $$LdW+WdL+CV=CdP_{C}+P_{C}dC+Dd\left(P_{D}+T_{D}\right)+\left(P_{D}+T_{D}\right)dD.$$

Therefore, $CV=-LdW+CdP_{C}+Dd\left(P_{D}+T_{D}\right)-\frac{\partial u}{\partial M}\frac{dM}{\lambda }$. Define MEB as the value of CV with respect to aggregate production $P_{Y}Y$. Using the implied terms $dP_{C}=dP_{D}=0$, we can have $MEB=-\left(1-\theta_{L}\right)\omega_{L}\tilde{W}+\omega_{D}\tilde{T_{D}}-\frac{u_{M}\omega_{M}}{\lambda }\tilde{M}$. As $\omega_{M}\tilde{M}=-\gamma_{E}\omega_{E}\tilde{E}-\gamma_{D}\omega_{D}\tilde{D}$, MEB can be rewritten as:

(13) $$MEB=-\left(1-\theta_{L}\right)\omega_{L}\tilde{W_{R}}+\frac{u_{M}}{\lambda }(\gamma_{D}\omega_{D}\tilde{D}+\gamma_{E}\omega_{E}\tilde{E}).$$

According to the government’s budget constraint, we can further have:

(14) $$\theta_{L}\omega_{L}\tilde{L}+\theta_{E}\omega_{E}\tilde{E}+\theta_{D}\omega_{D}\tilde{D}=-\theta_{L}\omega_{L}\tilde{W}-\omega_{E}\tilde{T_{E}}-\omega_{D}\tilde{T_{D}}-\omega_{L}\tilde{T_{L}}.$$

Substitute the zero-profit condition $0=\omega_{L}\left(\tilde{W}+\tilde{T_{L}}\right)+\omega_{E}\tilde{T_{E}}$ from Table 2, we can substitute the term $\tilde{T_{L}}$ in the above equation and obtain the following:

(15) $$\left(1-\theta_{L}\right)\omega_{L}\tilde{W_{R}}=\theta_{L}\omega_{L}\tilde{L}+\theta_{E}\omega_{E}\tilde{E}+\theta_{D}\omega_{D}\tilde{D}$$

#### 3.1.2. The Marginal Excess Burden (MEB) in the Form of “Distortion”

Based on the analysis above, MEB can be expressed as the sum of three types of distortions:

(16) $$MEB=-\theta_{L}\omega_{L}\tilde{L}-[\theta_{E}-\frac{u_{M}\gamma_{E}}{\lambda }]\omega_{E}\tilde{E}-[\theta_{D}-\frac{u_{M}\gamma_{D}}{\lambda }]\omega_{D}\tilde{E},$$

The first item on the right-hand side of Equation (16) represents the distortion of labor market led by labor income tax, while the second and the third one represent the distortion of labor market led by the environmental tax. If there is pre-distortion caused by the labor taxes in the economy, the increase in employment will improve social welfare. Labor taxes create a “wedge” between the marginal social benefits of employment as measured by extra outputs and the marginal opportunity costs of social opportunities as measured by abandoned leisure. In fact, more employment produces more output, which not only compensates workers for giving up their leisure, but also increases public income through taxes. The second and third terms on the right-hand side of Equation (16) represent environmental distortions (or environmental damage) caused by pollution in production and consumption, respectively. The welfare effects of the marginal growth of pollution are determined by the relative effects of tax and environmental factors: tax parameters $\theta_{D}$ and $\theta_{E}$ measure the marginal social benefits brought about by the increase of pollution and thus the increase of taxes. Environmental parameters $\frac{u_{M}\gamma_{E}}{\lambda }$ and $\frac{u_{M}\gamma_{D}}{\lambda }$ measure the marginal environmental damage caused by pollution in the process of production and consumption. If there are no environmental taxes in the initial equilibrium, that is, $\theta_{E}=\theta_{D}=0$, the reduction of pollution will improve overall welfare, because the marginal social benefits of pollution reduction exceed the marginal social costs.

In the optimal environment where there is no labor tax ($\theta_{L}=0$), the government does not need to raise public expenditure through distortionary taxes. The social opportunity cost of employment increase is just equal to its social income, so the change of employment will not affect social welfare. Equation (16) shows that the best environmental tax policy is to internalize externalities completely by satisfying the conditions of $\theta_{D}=\frac{u_{M}\gamma_{D}}{\lambda }$ and $\theta_{E}=\frac{u_{M}\gamma_{E}}{\lambda }$. Such an optimal environmental tax rate is known as the Pigouvian tax rate (the environment where distortionary labor taxes do not exist beforehand is called the “first-best environment”; the optimal environmental tax in “this first-best environment” is called “Pigouvian tax”). At the Pigouvian tax rate level, the welfare losses caused by increased pollution are in line with the welfare gains brought about by the expansion of the tax base, so that the marginal increase in demand for polluting inputs and polluting consumer goods will not affect social welfare through the channel of environmental distortions.

However, in the “second-best environment” with pre-existing distortionary labor tax ($\theta_{L}>0$), the marginal change in pollution may also affect social welfare through the channel of distorted labor market with employment affected. In the “second-best environment”, in which the government needs to finance public expenditure through distortionary labor taxes, the optimal environmental tax level usually deviates from the Pigouvian tax level. Whether a tax can improve social welfare depends not only on its impact on environmental distortions, but also on the feedback effect of employment [23,24]. If an environmental tax reform increases employment at the Pigouvian level, then the government’s best choice is to raise the environmental tax rate above the Pigouvian level. On the contrary, if raising the environmental tax rate above the Pigouvian level leads to a reduction in employment, then the best environmental tax policy is to set the tax rate below the Pigouvian level.

#### 3.1.3. The Marginal Excess Burden (MEB) in the Form of “Dividends”

MEB can also be expressed as the sum of various forms of dividends, as shown below:

(17) $$MEB=-\tilde{B}-\frac{u_{M}\omega_{M}}{\lambda }\tilde{M},$$

The first and second items on the right-hand side of Equation (17) represent “blue dividends” and “green dividends” respectively, where:

(18) $$\tilde{B}=\theta_{L}\omega_{L}\tilde{L}+\theta_{E}\omega_{E}\tilde{E}+\theta_{D}\omega_{D}\tilde{D},$$

$\tilde{B}$ is the tax base effect, representing the effects of environmental taxes on different tax combinations as financing means. The three items on the right-hand side of Equation (18) represent the tax base effect of labor taxes, environmental taxes on the firm, environmental taxes on the household consumption, respectively. If the tax base is eroded, the efficiency of the whole tax system as a means of financing is reduced. Equation (17) shows that MEB can be divided into tax base effect (the first item on the right-hand side of the equation) and environmental effect (the second item on the right-hand side of the equation). In fact, MEB represents two tasks of environmental tax reforms as well as the blue dividend and green dividend of the so-called “double dividend” hypothesis. A tax system, as a financing tool, should meet the demand for public expenditure at the lowest distortion cost. It should also focus on the internalization of environmental externalities. If environmental welfare is improved, a “green dividend” is obtained; if non-environmental welfare is improved, a “blue dividend” is obtained; if both the blue dividend and the green dividend are positive, a “double dividend” effect is realized.

Using the zero-profit condition $0=\omega_{L}\left(\tilde{W}+\tilde{T_{L}}\right)+\omega_{E}\tilde{T_{E}}$ and the government budget constraint condition $0=\omega_{L}\tilde{T_{L}}+\omega_{E}\tilde{T_{E}}+\omega_{D}\tilde{T_{D}}+\theta_{L}\omega_{L}\left(\tilde{W}+\tilde{L}\right)+\theta_{E}\omega_{E}\tilde{E}+\theta_{D}\omega_{D}\tilde{D}$, we can express the non-environmental welfare as:

(19) $$\tilde{B}=\left(1-\theta_{L}\right)\omega_{L}\tilde{W_{R}},$$

The right-hand side of Equation (19) indicates the effect of environmental taxes on the after-tax real wages of the family. From a non-environmental perspective, if the efficiency of the tax system is reduced, the real personal income will be reduced. The erosion of the tax base means that the tax rate must be increased to raise the same amount of public revenue. In order to raise the same amount of tax revenue, the government must raise the marginal tax rate, resulting in lower personal income. However, if the increase in environmental welfare outweighs the decline in the non-environmental dividend, the overall utility of the family may still be improved. In other words, even if the blue dividend is negative, the green dividend (environmental dividend) can be positive and large enough so that the total welfare level of the family will still be improved because of the environmental tax reform.

### 3.2. Effects of Environmental Taxes on the Household

#### 3.2.1. Simplified Solutions of the Model

The results based on logarithmic linearization in Table 2 are further simplified, as shown in Table 3. See Appendix D for the derivations.

The parameters of the simplified form of the solutions in the second and third columns of Table 3 represent the effects of tax changes on polluting consumer goods D and polluting input E, respectively. To study the effects of environmental taxes is to study the impacts of environmental taxes on environmental welfare and non-environmental welfare, or to study whether the environmental tax reform can achieve the “double dividend” effect, and whether they can reduce the distortion of the labor market. That is, we need to analyze how the environmental tax reform of fiscal-neutrality (in this paper, “environmental tax reforms of fiscal-neutrality” refers to the reform in which the tax burden is shifted from labor taxes to polluting inputs and polluting consumption goods, that is, the income from the environmental tax is used to finance the labor tax reduction, so that the government’s budget income remains unchanged) change the endogenous variables. Therefore, we next discuss how environmental tax reforms affect these endogenous variables from a specific initial equilibrium.

#### 3.2.2. Environmental Tax Reforms without Pre-existing Environmental Taxes in the Initial Equilibrium

As can be seen from the second column of Table 3, by the introduction of a low-rate environmental tax on households where the environmental tax revenue is used for labor tax relief, the employment and output will not be affected, as represented by $\theta_{D}=0$ in the first and second rows of Table 3. The levy of environmental taxes has no direct impacts on output, so the marginal output of labor and therefore the pre-tax wages and labor demand remain unchanged. Based on the zero-profit condition $0=\omega_{L}\left(\tilde{W}+\tilde{T_{L}}\right)+\omega_{E}\tilde{T_{E}}$, we can see that lower labor (value added) taxes will lead to the increase of market wage rates (the market wage rate W is the post-labor-tax and pre-consumption-tax wage). That is:

(20) $$\tilde{W}=-\tilde{T_{L}},$$

However, $\tilde{L_{S}}=\eta_{LL}\tilde{W_{R}}$ in Table 2 means that what affects labor supply incentives is the real after-tax wages $W_{R}$ (i.e., wages after VAT and indirect consumption taxes) rather than market wages W. Furthermore, environmental taxes create a “wedge” between these two types of wages. Therefore, the “wedge” between pre-tax and post-tax wages includes not only distortionary taxes on labor but also environmental taxes on consumption (as defined earlier, market wages W equals the pre-tax wages $W\left(1+T_{L}\right)$ deducted by the value-added tax on labor income, and after-tax wages $W_{R}$ equals the market wage W deducted by the consumption tax on pollution inputs). Whether the substitution of environmental taxes for labor taxes will increase real after-tax wages and thus stimulate labor supply depends on whether the effect of lower labor tax revenue can offset the effect of environmental tax increase.

Substituting (19) and (20) in $\tilde{W_{R}}=\tilde{W}-\frac{\omega_{D}}{\omega_{C}+\omega_{D}}\tilde{T_{D}}$ in Table 2, we can have:

(21) $$\tilde{T_{L}}+\frac{\omega_{D}}{\omega_{C}+\omega_{D}}\tilde{T_{D}}=-\frac{\tilde{B}}{\omega_{C}+\omega_{D}}$$

The right-hand side of Equation (21) reflects the tax base effect. If employment is not affected and there are no environmental taxes in the initial equilibrium, the tax base effect is zero. According to Equations (20) and (21), the effect of levying an environmental tax just offsets the effect of levying a lower labor tax on the “wedge” between the pre-tax and post-tax real wages. In fact, the two conditions of fiscal-neutrality and almost unchanged bases of consumption tax and labor tax make the environmental tax reforms substituting the labor income tax with environmental taxes. This only affects the “wedge” structure between pre-tax and post-tax wages, with little effects on the distortion of the whole tax system.

To sum up, if there are no environmental taxes in the initial equilibrium, the environmental tax reform will not affect employment, but it will improve the overall social welfare. Therefore, the employment double dividend effects of environmental taxes are absent, but the welfare double dividends effects may exist. However, the premise of this conclusion is that the change of environmental tax is sufficiently small.

#### 3.2.3. Environmental Tax Reforms with Pre-existing Environmental Taxes in the Initial Equilibrium

In order to study the impacts of large environmental tax reforms on employment and social welfare, we assume that environmental tax reforms begin with an initial equilibrium with environmental taxes. Our analysis shows that the effects of marginal changes of environmental taxes on employment depend on the wage elasticity of labor supply, the initial levels of environmental taxes, the substitution elasticity between contaminating and clean goods, and the size of the public sector.

The second and third rows in the second column of Table 3 show that when there is a positive environmental tax ($\theta_{D}>0$) in the initial equilibrium, if the uncompensated wage elasticity of labor supply ($\eta_{LL}$) is positive, the marginal increase of the environmental tax ($T_{D}$) levied on the polluting consumer goods D will reduce both employment and output levels. These negative effects are due to the reduced real after-tax wages thus the reduced labor supply incentives. And the reduced real after-tax wages are due to the fact that the reduction of labor tax does not fully compensate for the impacts of increased environmental taxes on real after-tax wages because of the eroded environmental tax base. Environmental tax increases will induce household use of clean consumer goods to replace polluting consumer goods. If the initial tax on contaminated consumer goods is positive, this behavioral response of the household will erode the tax base of the environmental tax, resulting in a negative tax base effect, namely $\tilde{B}<0$. Equation (21) shows that negative tax base effect will make the substitution of labor taxes with environmental taxes enlarge the “wedge” between pre-tax and post-tax wages. That is, $\tilde{T_{L}}+\frac{\tilde{T_{D}}\omega_{D}}{\omega_{C}+\omega_{D}}>0$. If the government wants to maintain fiscal-neutrality, it is impossible to substantially reduce the labor tax to offset the negative effect of increased environmental tax on real after-tax wages. As a result, lower marginal labor income will reduce labor supply, thereby reducing the level of employment and increasing unemployment. In other words, if environmental tax reforms begin with an initial equilibrium with pre-existing environmental taxes, a decline of real after-tax wages means that a “blue dividend” effect is often found to be false. Therefore, the fiscally neutral environment tax reform where the environment tax revenues fund for labor tax reduction will not improve the environmental quality while promoting employment and economic growth. In short, the double dividend effect does not exist.

In addition, the negative effects of environmental taxes on employment also depend on the initial levels of environmental taxes and the substitution elasticity between polluting and clean consumer goods, which also determine the level of real after-tax wages. Higher initial environmental taxes strengthen the erosion effect of tax base, which has a negative impact on income, and reduce the room for labor tax reduction. This has a negative impact on real post-tax wages, which also affects the incentives of labor supply. Higher substitution elasticity between contaminating and clean goods will strengthen the positive environmental effects of environmental taxes. However, it will also aggravate the negative impact on labor supply, thus further strengthening the erosion effect of tax base and limiting the room for tax reduction. Therefore, there is a trade-off between positive environmental effects and negative labor supply effects. The greater the environmental benefits of environmental taxes on polluting consumer goods, the greater the damage to labor supply incentives.

The size of the public sector, which is closely related to the public sector parameter Ω (Ω can be written as $\Omega =\left[\left(1-\theta_{L}\right)\omega_{L}-\theta_{D}\omega_{D}\right]-\eta_{LL}\omega_{G}>0$. The larger the proportion of public expenditure ($\omega_{G}$), the smaller Ω will be), also affects the employment effect of environmental taxes. The greater the government’s demand for public income and the higher the level of public expenditure, the greater the negative effects of environmental taxes on employment. This is because larger public sectors require higher initial labor taxes and environmental taxes (large $\theta_{L}$, $\theta_{E}$ and $\theta_{D}$) to raise revenues. However, environmental taxes as financing tools are of low efficiency. There are two effects of an increased environmental tax: one is to achieve more revenues from the same tax base, and the other is to erode the tax base. The financing efficiency of environmental taxes depends on the relative strength of these two effects.

The relative importance of the tax base effect to revenues depends on the initial tax rate and people’s behavioral responses and changes. The increase of the environmental tax rate can lead to two types of behavior responses. On the one hand, people’s use clean consumer goods instead of polluting consumer goods will erode the tax base of environmental tax, and this effect is determined by the initial environmental tax rate. On the other hand, the increase of the environmental tax will reduce employment, erode the environmental tax base, and reduce the efficiency of environmental taxes in raising funds. These two behavioral responses will not only reduce income, but also narrow the scope for tax reduction, impacting the labor supply negatively.

#### 3.2.4. Environmental Tax Reforms with Pre-existing Pigouvian Tax in the Initial Equilibrium

In the first-best environment, the government does not need to raise funds for public expenditure through distortionary taxes, that is $T_{L}=0$. Thus, the environmental taxes imposed on households should internalize the environmental externality completely, so the environmental tax rate should be $\theta_{D}=\frac{u_{M}\gamma_{D}}{\lambda }$. If the environmental tax rate is set at this level and the government only needs Pigouvian tax to finance public expenditure, the marginal reduction of the environmental tax will not affect overall welfare and there will be no distortions in the labor market ($T_{L}=0$). The impact of environmental deterioration and thus lowered environmental welfare caused by decreased environmental taxes is just offset by the positive effect of the expanded tax base. Under the first-best environment, the reduction of environmental taxes will increase labor supply and employment. However, if there is no pre-existing distortionary labor taxes ($T_{L}=0$), the increase in environmental taxes will not improve welfare level because the social opportunity cost of increased employment is just equal to the social benefits.

In a second-best environment, the government needs distortionary taxes to finance public expenditure. If the government reduces the environmental taxes below the Pigouvian tax, the overall welfare level will improve. Nonetheless, at the same time, the government must raise the labor tax $T_{L}$ to offset the effect of income reduction. In the first-best environment, the welfare gains of environmental improvement are equal to the welfare losses caused by tax base erosion. However, in the second-best environment, it is necessary to analyze how environmental taxes affect the total tax revenue through the effect of its own tax base and others. In the case of distortionary labor tax, substituting environmental taxes for labor taxes will erode the base of labor taxes because of reduced employment, and environmental taxes will reduce the ability of the entire tax system to collect other taxes. Therefore, reducing environmental taxes below Pigouvian tax and using the income to finance tax reduction on labor would reduce distortions associated with public expenditure and thus improve overall welfare. In other words, in the second-best environment, Pigouvian tax is not the best way to raise funds.

If the environmental tax rate falls below the Pigouvian level ($\theta_{D}=\frac{u_{M}\gamma_{D}}{\lambda }$), the welfare effect of raising the environmental tax rate is uncertain, because the increase in the environmental tax reduces not only pollution but also employment. To be more specific, higher environmental taxes will reduce environmental distortions and improve environmental welfare, but also aggravate the distortions of the labor market and reduce the level of employment. Therefore, environmental taxes do not necessarily lead to welfare improvement while reducing market distortion.

### 3.3. The Impact of Environmental Tax on the Firm

#### 3.3.1. Environmental tax Reforms without Environmental Taxes on Inputs in the Initial Equilibrium

If there is no input environmental tax ($\theta_{E}=0$) in the initial equilibrium, the parameters in the third column of Table 3 show that the environmental tax reform shifting the tax burden from labor income to pollution inputs has no effect on employment. The product tax reduces the demand for polluting inputs, resulting in lower domestic output. Therefore, if the employment level remains unchanged, labor productivity will inevitably decline, which in turn will lead to lower pre-tax wages. Based on the zero-profit condition $0=\omega_{L}\left(\tilde{W}+\tilde{T_{L}}\right)+\omega_{E}\tilde{T_{E}}$ in Table 2, the following can be obtained:

(22) $$\tilde{W}+\tilde{T_{L}}=-\frac{\omega_{E}}{\omega_{L}}\tilde{T_{E}}$$

Because of the international liquidity of pollution inputs, the environmental tax burden can be completely transferred to the labor force in the form of low productivity. Next, we will analyze the impact of environmental taxes on real post tax wages so as to determine its impact on labor supply. The impact of environmental taxes on real after-tax wages is the same as that on market wages, because there is no change in the environmental taxes levied on households. Equation (22) reflects that the impact of environmental tax on market wages depends on two aspects: the reduction of pre-tax wages caused by the increased environmental taxes and the reduction of labor taxes caused by the use of environmental tax revenue for tax reduction. Based on the government’s budget constraints in Table 2 and Equation (22), we can draw the following relationship between the reduction of labor taxes and the increase of environmental taxes on output:

(23) $$\tilde{T_{L}}+\frac{\omega_{E}}{\omega_{L}}\tilde{T_{E}}=-\tilde{B}$$

The right-hand side of Equation (23) is the tax base effect. If the initial environmental tax is 0, the employment will not be affected, and the tax base effect will be 0. Equations (22) and (23) show that after-tax wages are not affected by environmental taxes, and that fiscally-neutral environmental tax reforms will make the negative effect of the reduced pre-tax wages on after-tax wages offset the positive effect of the reduced labor taxes. In a small open economy, substituting input environmental taxes for labor tax is equivalent to substituting an implicit labor tax for an explicit labor tax.

When there is no environmental tax in the initial equilibrium, the introduction of low environmental taxes can improve the overall welfare. Environmental taxes have no effect on employment and labor market distortions, but they can reduce the level of pollution and thus improve the environment. Equation (22) also shows that implementing a low environmental tax would improve the environmental quality without compromising the ability of the entire tax system to raise funds, and thereby improve the welfare of the whole society. Hence, environmental tax reforms will help improve environmental quality and social welfare, but without obvious impacts on employment and tax efficiency. In short, there will not be “double dividend” effects.

#### 3.3.2. Environmental Tax Reforms with Environmental Taxes on Inputs in the Initial Equilibrium

The third row third column in Table 3 shows that the environmental tax reforms will reduce the level of employment when there are environmental taxes on polluting inputs ($T_{E}>0$) in the initial equilibrium, because the increase of environmental taxes will reduce real after-tax wages and reduce the willingness of labor supply. Environmental taxes reduce the demand for pollution inputs and reduce the marginal income of the labor force, affecting real labor income through two channels. On the one hand, environmental taxes reduce the marginal productivity of labor and pre-tax wages. On the other hand, raising environmental taxes erode the tax base, resulting in a negative tax base effect ($\tilde{B}<0$). Therefore, in order to maintain fiscal-neutrality, the government cannot reduce labor income tax to offset the negative impact of pre-tax wage decline. Equations (21) and (23) indicate that if the tax base of the initial environmental taxes is reduced ($\tilde{B}<0$), the after-tax wages will be reduced. The price of polluting inputs is determined by the world market, and the supply is of unlimited flexibility. The tax burden of environmental taxes is entirely borne by the labor factor, which is of non-liquidity, and is manifested in the form of reduced labor productivity. In fact, the environmental tax reform is to replace an explicit labor tax with a hidden one. As a financing tool, an explicit labor tax is more efficient than an implicit one, because an implicit one not only distorts the labor market but also reduces the demand for polluting input, and thus erodes the tax base. In other words, unlike the labor tax, the environmental tax distorts the production process.

The higher the initial tax rate, the greater the negative income effect caused by the decreased employment. The initial input environmental tax $\theta_{E}$ measures the gap between the social benefit and non-environmental costs of pollution. Therefore, the tax base effect of the environmental tax on pollution inputs measures the non-environmental costs associated with a clean environment, i.e., lower real wages and lower employment, will result in an increase in the overall burden of the tax system. If there is little room for firms to replace polluting inputs with labor ($\sigma_{LE}$ is small), the effects of environmental taxes on labor market distortions are small. However, the improvement of environmental welfare is also relatively small. Therefore, there is a trade-off between improving environmental quality and lowering public expenditure costs. Stated differently, replacing labor taxes with environmental taxes will not achieve a “double dividend” effect.

#### 3.3.3. Environmental Tax Reforms with Pigouvian Tax on Inputs in the Initial Equilibrium

If the initial environmental tax on input completely internalizes the environmental externalities, that is, $\theta_{E}=\frac{u_{M}\gamma_{E}}{\lambda }$, environmental tax reforms will not affect welfare through the channel of environmental distortions, so the welfare effects of environmental taxes are entirely determined by the distortions of the labor market. The parameters in the second row of Table 2 show that if the uncompensated wage elasticity of labor supply is greater than 0, the environmental tax reform starting with the Pigouvian tax rate will reduce the employment level. When there are distortionary taxes in the initial equilibrium of labor market, the reduction of employment will aggravate the distortions of labor market. The relative importance of environmental taxes to the labor market distortions depend on the overall income demand of the government. Therefore, a higher level of public expenditure will reduce the welfare benefits of environmental taxes instead of labor taxes. In fact, even if the initial environmental tax is lower than the Pigouvian tax level, this change in the tax system will damage social welfare. A substantial reduction in environmental taxes from Pigouvian level would reduce distortions in the labor market and thus improve social welfare.

## 4. Simulations

### 4.1. Data Description

Since the reform and opening up, China’s socialist market economic system has gradually improved. In the process of further opening up in the Western regions of China, Chongqing has continuously improved the level of economic opening. Consequently, Chongqing has been more effectively integrated into the overall domestic and international economic development, and its factor market has become more mature. The decisive role of market in resource allocation has been further improved. Theoretically, Chongqing can be regarded as a small open economy by ignoring other secondary imperfect competition factors in the market. Therefore, we use the macroeconomic data of Chongqing to simulate the conclusions above based on the analytic model.

For the scale problem involved in the model, we adopt the average approximate value of the data from 2012 to 2018 in the Chongqing Statistical Yearbook (2013–2019). For the proportions of labor and pollution input to the total regional economy, we use the ratios of urban and rural residents’ wages and energy consumption in recent years. According to the computing method of [25,26], the ratio of labor remuneration is about 0.46, while the perfect ratio is about 0.75, and the proportion of energy consumption is about 0.25. According to the Chongqing Statistical Yearbook 2013-2019, the ratio of consumption with respect to GDP during 2012–2018 is about 0.40, and the ratio of household consumption between contaminating products (i.e., products causing pollutions in their production and use) and clean products (i.e., products causing little pollution in their production and use) is about 1:3. Therefore, the ratio of contaminating products is 0.10 and the ratio of clean products is 0.30. For the proportion of government expenditure on public services, we use the ratio of total fiscal expenditure with respect to GDP, which is about 0.36 (between 2011 and 2018, Chongqing’s average fiscal revenue to GDP ratio is 36.16%). In terms of the tax parameters, the labor tax parameter is set to 0.36, which is the overall macro tax burden level of the family. In addition, because firms can reduce part of the tax burden by offsetting costs and deducting taxes, the environmental tax of households is higher than that of firms. Thus, the environmental tax parameters for households and firms are set as 0.2 and 0.25, respectively. To be more concrete, in the environmental tax parameter of labor income $\theta_{L}=\frac{T_{L}}{1+T_{L}}$, $T_{L}$ is represented by the ratio of the sum of individual income tax revenues of labor income and overall macro tax burden of labor service tax in value-added tax, with respect to GDP, and it equals about 0.25. This means $\theta_{L}=0.20$. For the environmental tax parameter of polluting products $\theta_{L}=\frac{T_{D}}{P_{D}+T_{D}}$, it is represented by the ratio of the sum of value-added tax and consumption tax on energy goods, with respect to GDP, and it equals about 0.25. According to [27] and [28], the substitution elasticity of labor and pollution inputs is estimated to be 0.4, the substitution elasticity between contaminants and cleaning products is 0.6, and the average elasticity of labor supply in China is about 3.48 (3.79 for urban areas about 2.00 for rural areas), which should also be close to the value in Chongqing. For clarity, the parameter setting of the model is listed in Table 4.

### 4.2. Simulation of the Model

Table 5 simulates the effect of a 10% increase in the environmental tax on contaminated inputs E and contaminated consumer goods D (since our model can only analyze the effect of marginal changes in the environmental tax, it is assumed that the environmental tax will be adjusted once; the marginal change of 10% of the environmental tax is too large in reality, however, our goal is to explain how the change of the environmental tax affects the levels of employment, polluting inputs, and consumer goods, and economic aggregate, etc.). The simulation results show that the 10% increase of household environmental tax can reduce the consumption of polluted goods by 5.25%, the total employment and economic output by 0.22%, and personal income by 0.22%. At the same time, the marginal increase in the environmental tax levied on households by 10% will also reduce the use of corporate polluting inputs and increase household consumption of clean goods by 1.75%. The increase of household environmental tax will make households consume more clean products. This can directly increase the consumption of clean products and indirectly reduce the amount of pollution inputs used by firms to produce polluted products. Therefore, the environmental tax reform on households has achieved a relatively large reduction in the consumption of pollutants at a relatively small economic cost. In other words, the environmental tax reform can play a role in environmental protection, improving the environmental quality and leading to an “environmental dividend” effect.

Furthermore, the simulation results show that a 10% increase of firm environmental tax (environmental tax imposed on polluted inputs) can reduce the consumption of polluted inputs by 8.64%. This is accompanied by a 1.23% decrease in employment, a 2.12% drop in household income, a 4.64% drop in aggregate economic volume, and a 4.55% drop in consumption. Hence, the reform of firm environmental tax has a significant rectification effect on firm behavior. It can greatly reduce the use of pollution inputs. However, it can negatively and significantly affect the total economic volume, household consumption of clean goods, as well as household income. Stated differently, the reform of firm environmental tax can achieve the “environmental dividend”, but not the “economic dividend” or the “double dividend”. There is a trade-off between the economic effect and environmental effect. In other words, the environmental tax reform on firms will not lead to a “double dividend”, as the benefit of achieving environmental quality will come with the sacrifice in the welfare of economic growth, employment, consumption and household income.

### 4.3. Simulation Results and Discussion

#### 4.3.1. The Environmental Effects of Environmental Taxes

The effect of environmental tax reform on environmental improvement is salient, but the improvement of environmental quality also requires paying the economic cost. Whether it is the reform of household or environmental tax on the firm, the marginal increase of environmental tax will directly inhibit the use of polluting goods or inputs, which is conducive to not only the improvement of environmental quality but also the cultivation of firms and households’ awareness of energy conservation and environmental protection. Therefore, the “environmental dividend” effect of environmental tax reform exists. However, environmental tax reform at the same time can sacrifice household income, employment and economic growth.

#### 4.3.2. Substitution Effect and Income Effect of Environmental Taxes

Family environmental tax has substitution effect and income effect, and the former is far greater than the latter. On the one hand, household environmental tax has negative but minimal impacts on household income and household purchasing power. On the other hand, the household environmental tax will affect household consumption behavior. Specifically, consumers will use clean consumer goods instead of polluting consumer goods. Generally speaking, the substitution effect of household environmental tax is much more significant, which is manifested by a very low reduction in household income but a substantial increase in clean goods consumption. In short, the household environmental tax can not only reduce pollution emissions and improving environmental quality, but also can guide households to change consumption behavior and cultivate people’s awareness of environmental protection.

The firm environmental tax has the substitution effect and the income effect. However, its substitution effect is weaker than its income effect. On the one hand, the levy of firm environmental tax will induce firms to use labor instead of polluting inputs. Nonetheless, as the elasticity of substitution between labor and pollution inputs is low, the substitution of labor for polluting inputs is rather limited. On the other hand, the firm environmental tax reduces the purchasing power of firms in two ways. First, the firm environmental tax has a negative impact on the total economic volume. As a result, the purchasing power of the whole economy shrinks, and the labor and polluting input factors purchased by firms decrease. Second, the family income and the associated purchasing power decrease. As a result, the quantity of pollutants purchased decreases. This can further lead to the reduction of market demand and indirectly affects the input needs of firms. In contrast, the substitution effect of firm environmental tax is insignificant because of the low substitution among factors. In short, the main effect of firm environmental tax is the income effect.

#### 4.3.3. Effects of Environmental Taxes on Employment and Income

Compared with the family environmental tax, the firm environmental tax has a greater impact on growth, employment and income. The reasons are as follow. Firstly, the family environmental tax is actually a direct tax. It is levied in the final consumption link, difficult to transfer, and has little impact on the production link. In contrast, the firm environmental tax is an indirect tax. It is levied in the production link, easy to transfer, and will create both factor market and product market. Therefore, compared with the family environmental tax, the firm environmental tax has a greater impact on the demand for labor factors and final income.

Secondly, the family and firm environmental tax has an indirect and direct impact on firm behavior, respectively. Moreover, the family environmental tax has a direct effect on consumers’ behavior. That is, it can motivate consumers to purchase clean products instead of polluting ones. It can also reduce household demand for consumer goods and thus reduce the market demand faced by firms. However, this negative effect due to consumer behavior changes can be absorbed. The firm environmental tax directly increases the marginal production costs of firms. In order to maximize profits, firms must reduce both labor input and polluting input. At the same time, as far as possible firm use labor instead of pollution input. However, this substitution is rather limited, resulting in a significant drop in polluting inputs and outputs.

#### 4.3.4. Effects of Environmental Taxes on Energy Saving and Emission Reduction

Compared with the household environmental tax, the firm environmental tax can more effectively achieve energy saving and emission reduction, but at a much higher economic cost. It is more effective if it is considered from the perspective of environmental protection only. On the other hand, the household environmental tax is better if the non-environmental perspective is also considered. Furthermore, because of its insignificant impact on employment and economic growth, the family environmental tax may encounter much less public resistance. In contrast, because the corporate environmental tax may significantly and negatively impact employment and economic growth, it may encounter the dual pressure from political and economic interest groups.

## 5. Conclusions and Policy Implications

In this paper, we first build a general equilibrium model for a small open economy and analyze the policy effects of environmental tax reform on environmental quality, social employment, economic growth, and family income. We then use the economic data of Chongqing municipality in China to conduct simulations test the theoretical model empirically. Our results demonstrate that there is a trade-off between the environmental effects and non-environmental effects of environmental tax reform. In other words, the “double dividend” effect of environmental tax reform does not exist. To be more specific, an increase of environmental tax rate will effectively reduce the use of polluting consumer goods by households and reduce the investment of enterprises in polluting factors. As a result, the “environmental dividend” can be achieved. However, an increase of environmental tax rate will have a negative impact on employment, family income and economic growth, indicating that the environmental tax reform does not generate “non-environmental dividend” effect. Moreover, the increase of environmental tax rate has both substitution effect and income effects on household consumption. Specifically, it can not only encourage households to substitute polluting consumer goods with clean ones, but also decrease the total consumption level of households.

Our findings based on model analysis and simulations have policy implications for potential environmental tax reforms in countries like China. Firstly, the basic starting point of environmental tax reform is environmental protection, not the “double dividend” or “blue dividend”. In other words, the conditions for the environmental tax reform to achieve double dividends are rather demanding. It is hence unrealistic or even counterproductive if the government pursues the “double dividend” effect and hopes that the environmental tax will solve the problems of unemployment and economic stagnation while alleviating the environmental, resource and energy problems. Secondly, the primary function of environmental tax is to alleviate environmental problems by motivating firms and households to change their behavior and reduce the use of polluting consumer goods and inputs, thereby improving environmental quality. Therefore, the government cannot expect a significant boost in terms of employment and economic growth through environmental tax reform. Third, the family environment tax and the corporate environmental tax should be levied simultaneously. The family environmental tax has a greater influence on the consumption of household polluting goods. And the firm environmental tax has a greater influence on the use of polluting inputs. Therefore, from the point of view of environmental protection, the two need to be introduced simultaneously. Although the negative effect of the firm environmental tax on economy is much greater than that of family environmental tax, the environmental quality cannot be sacrificed for the growth of economic indicators. A government should strive to achieve the coordinated and sustainable development of resources, environment and economy. Fourthly, in order to achieve more effective environmental protection, the government can levy higher environmental tax on households than firms. This is because the family environmental tax has less impact on employment and economic growth. Hence, it can achieve the same environmental protection goals at lower economic costs. Stated differently, from an economic point of view, higher environmental taxes on households can more effectively lower the cost of environmental tax reform. Moreover, the substitution effect of household environmental tax is larger than the income effect, while the substitution effect of firm environmental tax is smaller than the income effect. The household environmental tax can improve the environmental quality without causing excessive welfare loss to consumers, while the firm environmental tax needs consumers to pay more for the same degree of environmental quality improvement. Therefore, higher environmental taxes on households can actually protect consumer welfare to a greater extent.

In this paper, we make several assumptions, which can be relaxed in future research. For example, the assumption of a fully competitive market such as a small open economy excludes issues of incomplete competition. Furthermore, there are at least four future research directions. The first is to consider the incomplete competition market structure to be more realistic. The second is to consider the possibility of unbalanced government budget and analyze its impact on environmental tax reform. Third, in this paper, to make our model tractable and more focused to study the environmental tax and the “Double Dividend” hypothesis, we omit several factors issues such as exchange rate. Hence, in the future, it is worthwhile to consider factors like imports, exports, and exchange rate into the model. As such a model can be analytically intractable, numerical simulations might be primarily used. Last but not least, it is worthwhile to build a dynamic general equilibrium model to analyze the dynamic effect of environmental tax reform.

## Figures and Tables

**Table 1:** Variables, parameters, proportions and their relations.

**Basic Variables**	
Y = Output	L = Employment
E = Demands for the polluting inputs	C = Consumption of clean goods
D = Consumption of polluting goods	$L_{S}$ = Labor supply
W = Market wage	$W_{R}$ = Real post-tax wage
M = Environmental quality	G = Public consumption goods
$T_{L}$ = Ad valorem tax on labor incomes	$T_{E}$ = Quantity tax from polluting inputs
$T_{D}$ = Ad valorem tax on polluting consumption goods	P = Market prices of goods I(=Y,C,D,E,G)
**Parameters**	
$\sigma_{LE}$ = Labor’s substitution elasticity for pollution input	$\sigma_{CD}$ = Substitution elasticity of clean goods C for polluting goods D
$\sigma_{V}$ = Substitution elasticity of leisure for personal consumption	$\eta_{LL}=V\left(\sigma_{V}-1\right)$ = Uncompensated labor supply elasticity
$\gamma_{E}=-\frac{m_{E}}{P_{E}+T_{E}}$	$\gamma_{D}=-\frac{m_{D}}{P_{D}+T_{D}}$
**Proportions**	
$\omega_{L}=\frac{\left(1+T_{L}\right)WL}{P_{Y}Y}$	$\omega_{E}=\frac{\left(P_{E}+T_{E}\right)E}{P_{Y}Y}$
$\omega_{C}=\frac{P_{C}C}{P_{Y}Y}$	$\omega_{D}=\frac{\left(P_{D}+T_{D}\right)D}{P_{Y}Y}$
$\omega_{G}=\frac{P_{G}G}{P_{Y}Y}$	$\omega_{M}=\frac{M}{P_{Y}Y}$
**Tax parameters**	
$\tilde{T_{L}}=\frac{dT_{L}}{1+T_{L}}$	$\tilde{T_{L}}=\frac{dT_{E}}{P_{E}+T_{E}}$
$\tilde{T_{L}}=\frac{dT_{D}}{P_{D}+T_{D}}$	$\theta_{L}=\frac{T_{L}}{1+T_{L}}$
$\theta_{E}=\frac{T_{E}}{P_{E}+T_{E}}$	$\theta_{D}=\frac{T_{D}}{P_{D}+T_{D}}$
**Proportional relations**	
$\omega_{E}+\omega_{L}=1$	$\left(1-\theta_{L}\right)\omega_{L}=\omega_{C}+\omega_{D}$
$\omega_{G}=\theta_{L}\omega_{L}+\theta_{E}\omega_{E}+\theta_{D}\omega_{D}$	$1=\omega_{C}+\omega_{G}+\left(1-\theta_{E}\right)\omega_{E}+\left(1-\theta_{D}\right)\omega_{D}$

**Table 2:** Linear logarithm of the model.

**Firm**	
Domestic output	$\tilde{Y}=\omega_{E}\tilde{E}+\omega_{L}\tilde{L}$
Demand for polluting input	$\tilde{E}=\tilde{L}-\sigma_{LE}\left[\tilde{T_{E}}-\left(\tilde{W}+\tilde{T_{L}}\right)\right]$
Zero profit condition	$0=\omega_{E}\left(\tilde{W}+\tilde{T_{L}}\right)+\omega_{E}\tilde{T_{E}}$
**Household**	
Household budget constraint	$\omega_{L}\left(1-\theta_{L}\right)\left(\tilde{L}+\tilde{W}\right)=\omega_{C}\tilde{C}+\omega_{D}\left(\tilde{D}+\tilde{T_{D}}\right)$
Labor supply	$\tilde{L_{S}}=\eta_{LL}\tilde{W_{R}}$
Real after-tax wage	$\tilde{W_{R}}=\tilde{W}-\frac{\omega_{D}}{\omega_{D}+\omega_{C}}\tilde{T_{D}}$
Individual consumption	$\tilde{C}-\tilde{D}=\sigma_{CD}\tilde{T_{D}}$
**Government**	
Government budget constraint	$0=\omega_{L}\tilde{T_{L}}+\omega_{E}\tilde{T_{E}}+\omega_{D}\tilde{T_{D}}+\theta_{L}\omega_{L}\left(\tilde{W}+\tilde{L}\right)+\theta_{E}\omega_{E}\tilde{E}+\theta_{D}\omega_{D}\tilde{D}$
Equilibrium of labor market	$\tilde{L_{S}}=\tilde{L}$
Environmental quality	$\omega_{M}\tilde{M}=-\gamma_{E}\omega_{E}\tilde{E}-\gamma_{D}\omega_{D}\tilde{D}$
Material balance	$\tilde{Y}=\omega_{C}\tilde{C}+\left(1-\theta_{D}\right)\omega_{D}\tilde{D}+\left(1-\theta_{E}\right)\omega_{E}\tilde{E}$
**Variable description**	
Endogenous variables	$\tilde{Y},\tilde{L},\tilde{E},\tilde{D},\tilde{C},\tilde{L_{S}},\tilde{W},\tilde{W_{R}},\tilde{M},\tilde{T_{L}}$
Exogenous variables	$\tilde{T_{D}},\tilde{T_{E}},\tilde{G}=0$
	$\tilde{P_{Y}}=\tilde{P_{E}}=\tilde{P_{D}}=\tilde{P_{C}}=\tilde{P_{G}}=0$

**Table 3:** Simplified solutions of the model.

	$\tilde{T_{D}}$	$\tilde{T_{E}}$
$\Omega \tilde{Y}$	$-\eta_{LL}\theta_{D}\omega_{C}\omega_{D}\frac{\sigma_{CD}}{\omega_{C}+\omega_{D}}$	$-\left(\Omega +\theta_{E}\eta_{LL}\right)\omega_{E}\frac{\sigma_{LE}}{\omega_{L}}$
$\Omega \tilde{L}$	$-\eta_{LL}\theta_{D}\omega_{C}\omega_{D}\frac{\sigma_{CD}}{\omega_{C}+\omega_{D}}$	$-\eta_{LL}\theta_{E}\omega_{E}\frac{\sigma_{LE}}{\omega_{L}}$
$\Omega \tilde{E}$	$-\eta_{LL}\theta_{D}\omega_{C}\omega_{D}\frac{\sigma_{CD}}{\omega_{C}+\omega_{D}}$	$-\left(\Omega +\theta_{E}\omega_{E}\eta_{LL}\right)\frac{\sigma_{LE}}{\omega_{L}}$
$\Omega \tilde{D}$	$-\left[\Omega +\theta_{D}\omega_{D}\left(1+\eta_{LL}\right)\right]\omega_{C}\frac{\sigma_{CD}}{\omega_{C}+\omega_{D}}$	$-\left(1+\eta_{LL}\right)\theta_{E}\omega_{E}\frac{\sigma_{LE}}{\omega_{L}}$
$\Omega \tilde{C}$	$-\left[\Omega +\theta_{D}\omega_{D}\left(1+\eta_{LL}\right)\right]\omega_{D}\frac{\sigma_{CD}}{\omega_{C}+\omega_{D}}$	$-\left(1+\eta_{LL}\right)\theta_{E}\omega_{E}\frac{\sigma_{LE}}{\omega_{L}}$
$\Omega \tilde{B}$	$-\theta_{D}\omega_{C}\omega_{D}\sigma_{CD}$	$-\left(1-\theta_{L}\right)\theta_{E}\omega_{E}\frac{\sigma_{LE}}{\omega_{L}}$
$\Omega =\left[\left(1-\theta_{L}\right)\omega_{L}-\theta_{D}\omega_{D}\right]-\eta_{LL}\left(\theta_{L}\omega_{L}+\theta_{E}\omega_{E}+\theta_{D}\omega_{D}\right)>0$		

**Table 4:** Model parameters for simulation.

Proportion	Tax	Elasticity
$\omega_{L}=0.54$	$\theta_{L}=0.36$	$\sigma_{LE}=0.4$
$\omega_{E}=0.46$	$\theta_{E}=0.2$	$\sigma_{CD}=0.6$
$\omega_{D}=0.10$	$\theta_{D}=0.25$	$\sigma_{V}=3.48$
$\omega_{C}=0.30$	-	V=0.15
$\omega_{G}=0.36$	-	$\eta_{LL}=0.37$

**Table 5:** Economic effects of 10% increase in environmental taxes (unit: %).

	10% Increase on Household Environment Tax	10% Increase on Firm Environment Tax
Impact on total economic volume	−0.22	−4.64
Impact on employment	−0.22	−1.23
Impact on polluting inputs by firms	−0.22	−8.64
Impact on consumption of polluting goods by households	−5.25	−4.55
Impact on consumption of clean goods by households	1.75	−4.55
Impact on household income	−0.22	−2.12

## Appendix A. Processing of Firm’s Function

We can derive from the firm’s production function with constant return to scale that $\tilde{Y}=\omega_{E}\tilde{E}+\omega_{L}\tilde{L}$. The firm’s production function Y=F(E,L) with constant returns to scale, is first order homogeneous to E and L, thus YL=F(EL,1). Let y=YL,e=EL, we have y=f(e). In fact, this is the firm’s per capita production function, where the lowercase letter represents per capita variable. The first conditions for maximizing profit are: $\frac{W\left(1+T_{L}\right)}{P_{Y}Y}=\frac{\partial Y}{\partial L}=f-ef^{\prime }$ and $\frac{P_{E}+T_{E}}{P_{Y}Y}=\frac{\partial Y}{\partial E}=f^{\prime }$. Then we can obtain ∂Y∂E∂Y∂L=PE+TEW(1+TL)=f′f−ef′. When logarithmic linearization is applied to this formula and the formula $\tilde{P_{Y}}=\tilde{P_{E}}=0$ is utilized, we can have:

(A1) ∂(∂Y∂E∂Y∂L)(∂Y∂E∂Y∂L)=PE˜+TE˜−W˜−TL˜=ef″f′ff−ef′e˜.

The substitution elasticities of polluting input *E* and labor *L* satisfy

(A2) 1σLE=−∂(∂Y∂E∂Y∂L)∂(EL)EL∂Y∂E∂Y∂L=−∂(f′f−ef′)∂e=−ef″f′ff−ef′

Then we can obtain $\tilde{W}+\tilde{T_{L}}-\tilde{T_{E}}=\frac{1}{\sigma_{LE}}\tilde{e}=\frac{1}{\sigma_{LE}}\left(\tilde{E}-\tilde{L}\right)$, which can be rewritten as $\tilde{E}=\tilde{L}-\sigma_{LE}\left[\tilde{T_{E}}-\left(\tilde{W}+\tilde{T_{L}}\right)\right]$, recessively determining the demand for polluting input factor *E* under the provided employment level, with the second variable $\tilde{L}$ denoting the substitution effect caused by the relative changes of prices. Take the logarithmic linearization of the zero-profit condition of the firm, we can obtain $0=\omega_{L}\left(\tilde{W}+\tilde{T_{L}}\right)+\omega_{E}\tilde{T_{E}}$.

## Appendix B. Processing of Household Function

We first derive the labor supply function. Defined Q=q(C,D) as the combined consumption function, where Q can be regarded as the weighted average of C and D, and $P_{Q}$ is the price of the combined consumption goods, the weighted average of $P_{C}$ and $P_{D}+T_{D}$. As the problem of maximizing family utility becomes $max\;U=u\left[g\left(V,Q\right),h\left(G,M\right)\right],\;st.W\left(1-V\right)=P_{Q}$, the first-order conditions are ∂u∂Q=−λPQ,∂u∂V=−λW→∂u∂Q∂u∂V=PQW. According to the first-order conditions of the family utility function maximization, we can have ∂u∂Q∂u∂V=PQW, indicating that PQ˜−W˜=∂(∂u∂Q∂u∂V)∂u∂Q∂u∂V. As the substitution elasticity leisure with respect to private consumer goods $\sigma_{V}$ satisfies 1∂V=−∂(∂u∂Q∂u∂V)∂u∂Q∂u∂VQV∂(QV), we can have $\tilde{P_{Q}}-\tilde{W}=\frac{1}{\partial V}\left(\tilde{Q}-\tilde{V}\right)$. According to the definitions of Q and $P_{Q}$, we have $Q=\frac{\omega_{C}}{\omega_{C}+\omega_{D}}C+\frac{\omega_{D}}{\omega_{C}+\omega_{D}}D$, $P_{Q}=\frac{\omega_{C}}{\omega_{C}+\omega_{D}}P_{C}+\frac{\omega_{D}}{\omega_{C}+\omega_{D}}\left(P_{D}+T_{D}\right)$.

As $\tilde{P_{C}}=\tilde{P_{D}}=0$, we can obtain $\tilde{Q}=\frac{\omega_{C}}{\omega_{C}+\omega_{D}}\tilde{C}+\frac{\omega_{D}}{\omega_{C}+\omega_{D}}\tilde{D},\;\mathrm{and}\;\tilde{P_{Q}}=\frac{\omega_{D}}{\omega_{C}+\omega_{D}}\tilde{T_{D}}$. The household’s budget constraint is $WL_{s}=P_{C}C+P_{D}\left(P_{D}+T_{D}\right)$. With $L=L_{s}$, we can have $WL=P_{Q}Q=P_{C}C+P_{D}\left(P_{D}+T_{D}\right)$. By logarithmic linearization, we can have $\tilde{W}+\tilde{L}=\frac{\omega_{D}}{\omega_{C}+\omega_{D}}\tilde{T_{D}}+\frac{\omega_{C}}{\omega_{C}+\omega_{D}}\tilde{C}+\frac{\omega_{D}}{\omega_{C}+\omega_{D}}\tilde{D}$. Thus, $\left(\omega_{C}+\omega_{D}\right)\left(\tilde{W}+\tilde{L}\right)=\omega_{C}\tilde{C}+\omega_{D}\left(\omega_{D}+\tilde{T_{D}}\right)$.

As $\omega_{C}+\omega_{D}=\omega_{L}\left(1-\theta_{L}\right)$, we have $\left(\omega_{C}+\omega_{D}\right)\left(\tilde{W}+\tilde{L}\right)=\omega_{L}\left(1-\theta_{L}\right)\left(\tilde{W}+\tilde{L}\right)=\omega_{C}\tilde{C}+\omega_{D}\left(\omega_{D}+\tilde{T_{D}}\right)$. According to the definitions of Q and $P_{Q}$, together with $\tilde{P_{C}}=\tilde{P_{D}}=0$, we can have the followings: $\tilde{Q}=\frac{\omega_{C}}{\omega_{C}+\omega_{D}}\tilde{C}+\frac{\omega_{D}}{\omega_{C}+\omega_{D}}\tilde{D},\tilde{P_{Q}}=\frac{\omega_{D}}{\omega_{C}+\omega_{D}}\tilde{T_{D}}$.

As $\omega_{L}\left(1-\theta_{L}\right)\left(\tilde{W}+\tilde{L}\right)=\omega_{C}\tilde{C}+\omega_{D}\left(\omega_{D}+\tilde{T_{D}}\right)$, we can then obtain $\tilde{Q}=\tilde{L}+\tilde{W_{R}}$. Substituting the expressions of Q and $P_{Q}$ into $\tilde{P_{Q}}-\tilde{W}=\frac{1}{\partial V}\left(\tilde{Q}-\tilde{V}\right)$, we have $\tilde{V}-\tilde{L}=\left(1-\sigma_{V}\right)\tilde{W_{R}}$. According to the definitions of V and $\eta_{LL}$, $\tilde{V}=-\frac{L}{V}\tilde{L}$ and $\eta_{LL}=1-\sigma_{V}$. Substituting these two expressions in the equation above, we can get $\tilde{L_{S}}=\eta_{LL}\tilde{W_{R}}$.

Based on the relationship between the real post tax wage and market wage, we can obtain $\tilde{W_{R}}=\tilde{W}-\frac{\omega_{D}}{\omega_{C}+\omega_{D}}\tilde{T_{D}}$ by logarithmic linearization. $\sigma_{CD}$, the elasticity of substitution between the personal consumption goods C and D, satisfies 1σCD=−∂(∂u∂D∂u∂C)∂(DC)DC∂u∂D∂u∂C. Divide Equation (6) by Equation (7), we can have ∂u∂D∂u∂C=PD+TDPC, from which we can obtain the following by logarithmic linearization using the equations $\tilde{P_{C}}=\tilde{P_{D}}=0$ and 1σCD=−∂(∂u∂D∂u∂C)∂(DC)DC∂u∂D∂u∂C. That is, TD˜=∂(∂u∂D∂u∂C)∂u∂D∂u∂C=−1σCD(D˜−C˜), and thus $\tilde{C}-\tilde{D}=\sigma_{CD}\tilde{T_{D}}$.

## Appendix C. Processing of Government Function

Using logarithmic linearization of the government’s budget constraint and the condition $\tilde{P_{E}}=\tilde{P_{D}}=\tilde{P_{G}}=\tilde{G}=0$, we can obtain $0=\omega_{L}\tilde{T_{L}}+\omega_{E}\tilde{T_{E}}+\omega_{D}\tilde{T_{D}}+\theta_{L}\omega_{L}\left(\tilde{W}+\tilde{L}\right)+\theta_{E}\omega_{E}\tilde{E}+\theta_{D}\omega_{D}\tilde{D}$. As the labor market is assumed to be clear, $L=L_{S}$. Therefore, $\tilde{L_{S}}=\tilde{L}$. By logarithmic linearization of the environmental quality function, we can have $\omega_{M}\tilde{M}=\frac{m_{E}}{P_{E}+T_{E}}\omega_{E}\tilde{E}+\frac{m_{D}}{P_{D}+T_{D}}\omega_{D}\tilde{D}$. Letting $\gamma_{E}=-\frac{m_{E}}{P_{E}+T_{E}}$ and $\gamma_{D}=-\frac{m_{D}}{P_{D}+T_{D}}$, we can have $\omega_{M}\tilde{M}=-\gamma_{E}\omega_{E}\tilde{E}-\gamma_{D}\omega_{D}\tilde{D}$. And by logarithmic linearization of the equation of international balance of payments equation, we can have $\tilde{Y}=\omega_{C}\tilde{C}+\left(1-\theta_{D}\right)\omega_{D}\tilde{D}+\left(1-\theta_{E}\right)\omega_{E}\tilde{E}$.

## Appendix D. The Solution of the Simplified Model

Substituting $\tilde{Y}=\omega_{C}\tilde{C}+\left(1-\theta_{D}\right)\omega_{D}\tilde{D}+\left(1-\theta_{E}\right)\omega_{E}\tilde{E}$ with $\tilde{Y}=\omega_{E}\tilde{E}+\omega_{L}\tilde{L}$, we can eliminate the term $\tilde{Y}$ and obtain $\omega_{L}\tilde{L}=-\theta_{E}\omega_{E}\tilde{E}+(1-\theta_{D})\omega_{D}\tilde{D}+\omega_{C}\tilde{C}$, in which the three endogenous variables can be substituted with the expression $\tilde{E}=\tilde{L}-\sigma_{LE}\left[\tilde{T_{E}}-\left(\tilde{W}+\tilde{T_{L}}\right)\right]$. Also, substituting $0=\omega_{L}\left(\tilde{W}+\tilde{T_{L}}\right)+\omega_{E}\tilde{T_{E}}$ with, we can have $\tilde{E}=\tilde{L}-\frac{\sigma_{LE}}{\omega_{L}}\tilde{T_{E}}$.

We can establish the simultaneous equations $\tilde{W_{R}}=\tilde{W}-\frac{\omega_{D}}{\omega_{C}+\omega_{D}}\tilde{T_{D}}$, $\omega_{L}(1-\theta_{L})\left(\tilde{L}+\tilde{W}\right)=\omega_{C}\tilde{C}+\omega_{D}(\tilde{D}+\tilde{T_{D})}$ and $\tilde{C}-\tilde{D}=\sigma_{CD}\tilde{T_{D}}$. We solve them and have: as follows:

$$\tilde{C}=\tilde{L}+\tilde{W_{R}}+\frac{\omega_{D}}{\omega_{C}+\omega_{D}}\sigma_{CD}\tilde{T_{D}},\;\mathrm{and}\;\tilde{D}=\tilde{L}+\tilde{W_{R}}-\frac{\omega_{D}}{\omega_{C}+\omega_{D}}\sigma_{CD}\tilde{T_{D}}.$$

Substitute the expressions of $\tilde{C}$, $\tilde{D}$ and $\tilde{E}$ into the first equation above, we can obtain

(A3) $$[\theta_{L}\omega_{L}+\theta_{E}\omega_{E}+\theta_{D}\omega_{D}]\tilde{L}=\left[\left(1-\theta_{L}\omega_{L}\right)-\theta_{D}\omega_{D}\right]\tilde{W_{R}}+\theta_{E}\omega_{E}\frac{\sigma_{LE}}{\omega_{L}}\tilde{T_{E}}+\theta_{D}\omega_{C}\omega_{D}\frac{\sigma_{CD}}{\omega_{C}+\omega_{D}}\tilde{T_{D}},$$

where we use the proportion equation $\omega_{L}(1-\theta_{L})=\omega_{C}+\omega_{D}$. Combined with the labor supply function $\tilde{L_{S}}=\eta_{LL}\tilde{W_{R}}$, the above equation can be rewritten in the form of the following matrix:

(A4) $$\left(\begin{array}{cc} \theta_{L}\omega_{L}+\theta_{E}\omega_{E}+\theta_{D}\omega_{D} & -\left[\left(1-\theta_{L}\omega_{L}\right)-\theta_{D}\omega_{D}\right]\\ 1 & -\eta_{LL} \end{array}\right)\left(\begin{array}{c} \tilde{L}\\ \tilde{W_{R}} \end{array}\right)=\left(\begin{array}{cc} \theta_{D}\omega_{C}\omega_{D}\frac{\sigma_{CD}}{\omega_{C}+\omega_{D}} & \theta_{D}\omega_{E}\frac{\sigma_{LE}}{\omega_{L}}\\ 0 & 0 \end{array}\right)\left(\begin{array}{c} \tilde{T_{D}}\\ \tilde{T_{E}} \end{array}\right).$$

By transposing the matrix on the left side of the equation, the endogenous variables can be solved as:

(A5) $$\Omega \left(\begin{array}{c} \tilde{L}\\ \tilde{W_{R}} \end{array}\right)=\left(\begin{array}{cc} -\eta_{LL} & \left[\left(1-\theta_{L}\omega_{L}\right)-\theta_{D}\omega_{D}\right]\\ -1 & \theta_{L}\omega_{L}+\theta_{E}\omega_{E}+\theta_{D}\omega_{D} \end{array}\right)\left(\begin{array}{cc} \theta_{D}\omega_{C}\omega_{D}\frac{\sigma_{CD}}{\omega_{C}+\omega_{D}} & \theta_{D}\omega_{E}\frac{\sigma_{LE}}{\omega_{L}}\\ 0 & 0 \end{array}\right)\left(\begin{array}{c} \tilde{T_{D}}\\ \tilde{T_{E}} \end{array}\right).$$

In the above equation, $\Omega =\left[\left(1-\theta_{L}\omega_{L}\right)-\theta_{D}\omega_{D}\right]-\eta_{LL}\left(\theta_{L}\omega_{L}+\theta_{E}\omega_{E}+\theta_{D}\omega_{D}\right)$. Only when Ω>0 can the stability be ensured. Now, we can calculate the solution of $\tilde{L}$ and $\tilde{W_{R}}$ from the above equation:

(A6) $$\Omega \tilde{W_{R}}=\theta_{D}\omega_{C}\omega_{D}\frac{\sigma_{CD}}{\omega_{C}+\omega_{D}}\tilde{T_{D}}-\theta_{D}\omega_{E}\frac{\sigma_{LE}}{\omega_{L}}\tilde{T_{E}}.$$

Substituting the above equations into the first three equations of Table 2, we can obtain the simplified solutions of production, household consumption and polluting input, as is shown in Table 3.
